# Summer shifts of bacterial communities associated with the invasive brown seaweed *Sargassum muticum* are location and tissue dependent

**DOI:** 10.1371/journal.pone.0206734

**Published:** 2018-12-05

**Authors:** Alexandra Serebryakova, Tania Aires, Frédérique Viard, Ester A. Serrão, Aschwin H. Engelen

**Affiliations:** 1 Center for Marine Sciences (CCMAR), F.C.T. University of Algarve, Faro, Portugal; 2 Sorbonne Université, CNRS, UMR 7144 AD2M, Station Biologique de Roscoff, UPMC Univ Paris, Roscoff, France; Oklahoma State University, UNITED STATES

## Abstract

Seaweed-associated microbiota experience spatial and temporal shifts in response to changing environmental conditions and seaweed physiology. These shifts may result in structural, functional and behavioral changes in the host with potential consequences for its fitness. They, thus, may help the host to adapt to changing environmental conditions. The current knowledge of seasonal variation of seaweed-associated microbiota is however still limited. In this study, we explored temporal and spatial variation of microbial communities associated with the invasive brown seaweed *S*. *muticum*. We sampled in northern and southern Portugal, in September, March and July-August (summer). In addition, as (pseudo-)perennial seaweeds display seasonal reproductive phenology, we sampled various parts of the individuals to disentangle the effect of temporal changes from those due to structural development variations. The diversity and structure of associated microbial communities were determined using next generation sequencing of the variable regions V5-7 of the 16S rDNA. We expected to find differentiation in associated microbial communities between regions and sampling months, but with differences depending on the seaweed structure examined. As expected, the study revealed substantial temporal shifts in *S*. *muticum* microbiome, for instance with large abundance of *Rhodobacteraceae* and *Loktanella* in September-March but prevalence of *Pirellulales* during the summer months. Variations between regions and tissues were also observed: in northern Portugal and on basal structures, bacterial diversity was higher as compared to the South and apical parts. All examined seaweed structures showed temporal differences in associated microbial community structure over time, except for holdfasts between September and March. Bacteria contributing to these changes varied spatially. Conversely to all other structures, the holdfast also did not show differences in associated community structure between southern and northern regions. Our study highlights the importance of structural microscale differentiations within seaweeds hosts with regard to their associated microbial communities and their importance across temporal and spatial dimensions.

## Introduction

Seaweeds live in association with abundant and diverse microbiota, which plays an important role in the life of its hosts in nature [1, 2, 3]. Seaweeds rely on associated microbial communities for diverse functions including morphological development [4, 5, 6, 7, 8], consumption of organic matter and nitrogen source [9], defense [10, 11, 12, 13, 14, 15, 16], or provision of vitamins [17]. Microbial host-specificity has been reported (e.g., in fucoid seaweed *Phyllospora comosa* [18]) but most studies documented changes of seaweed associated microbial communities in response to various factors and conditions [11, 18].

Structure and composition of the seaweed-associated microbiota are known to change with the host conditions, as well as in space and time [11, 18, 19, 20, 21, 22, 23, 24, 25, 26, 27]. Regarding host condition, for example, healthy vs. stressed *Ecklonia radiata* exhibit different microbial communities [28]. Host traits may therefore be critical in determining the community structure of associated microbiota or *vice versa*. Other studies documented spatial variation [29], for instance, high within-species variability was observed among microbial communities associated with specimens of *Ulva australis* from different rock pools [30]. However, most studies documented shifts in microbial communities over time, varying from short-term (i.e. less than a day) to long-term (i.e. inter-annual) scales, with changes most often associated with seasonal variability [21, 31]. Some bacterial taxa seem more representative of a given season than others [19, 21, 22, 25]. For instance, bacterial communities associated with *Fucus vesiculosus* showed persistent seasonal variation, at the phyla level, over two consecutive years [21]. Recent research conducted on the Mediterranean seaweed *Cystoseira compressa* also revealed rather dynamic associated bacterial communities [22]. Bacterial communities associated with thalli of *C*. *compressa* displayed a clear successional pattern over time as well as an increase in abundance of pathogenic bacteria, associated with natural degradation of thalli at the end of the annual life cycle [22].

Seasonal variations are thought to be related to the combined effect of biotic (i.e. seaweed growth cycle, the age of the algal tissue) and abiotic factors (i.e. seawater temperature) [19, 22, 25]. *Planctomycetes*, *Verrucomicrobia* and *Alphaproteobacteria*, for example, were among the early colonizers of young thalli in *Laminaria hyperborea* [19]. As the algal tissue aged and biofilms matured, communities associated with *L*. *hyperborea* were complemented by *Gammaproteobacteria*, *Bacteroidetes*, and *Cyanobacteria* [19]. Other biotic factors include interactions (including competition) among bacterial taxa (i.e. both internal and from the surrounding water; [19]), consequences of biological interactions with organisms from other trophic levels (i.e. grazing, cross-feeding) as well as the loss of certain functions by bacteria (which results in the dependence on services provided by other microorganisms, [32]). Concerning abiotic factors, seasonal shifts in microbial community composition generally mirror seasonal environmental changes [31]. In particular, the summer increase of water temperature is likely to modify bacterial community composition [19, 22]. During summer, microbial communities on *L*. *hyperborea* were for instance characterized by a high diversity and such conditions were thought to be beneficial for seaweed-associated bacteria populating algal exudates [19]. On the other hand, some bacteria, such as *Betaproteobacteria* and *Verrucomicrobia* associated with *L*. *hyperborea*, were only observed in months when the seawater temperature was below 10°C, suggesting a preference for colder seawater temperatures [19]. Climate change and ocean acidification are also expected to intensify these changes [33, 34, 35].

Apart from the seasonality, host-associated microbiota is documented to be tissue-specific [24]. While microbial tissue-specificity was studied previously in, for example, corals [36], the number of studies conducted on seaweeds is very limited, but suggest that different seaweed tissues are populated by specific bacteria [24]. For instance, *Laminaria saccharina* had specific bacteria within its young and undisturbed tissues regardless of seasonality or geographic location [24]. It still remains to be explored to what extent tissue differentiation is affected seasonally. In addition, although many studies explored the seasonal differences of seaweed-associated microbiota and surrounding water column [19, 21, 22, 30, 37], no studies looked at similarities between the seaweed microbiome and surrounding water over time and, more importantly, at tissue or structure specificity. Differences in microbial communities of a seaweed between summer and winter can be directly related to its development and the structures sampled during each time. In winter, for example, pseudo-perennial seaweeds (as *S*. *muticum*) might consist of only a basal holdfast whereas in early summer they might be several meters long with various different structures present including blades, floatation vesicles and reproductive structures (receptacles), which each might have a different associated microbiome.

The present study addresses the disentanglement of spatial and temporal shifts in seaweed associated bacterial communities among different seaweed structures using the pseudo-perennial invasive brown seaweed *Sargassum muticum* as a case study. This brown alga is one of the most invasive macroalgae in the northern hemisphere, but its invasive success is not yet entirely understood [38]. Microbiota might play a key role in the acclimatization of this non-native seaweed, but it has been so far poorly examined. As detailed before, several studies indeed showed that bacterial seasonal variations are driven by environmental factors, notably seawater temperature. Seawater temperature data from southern to northern Portugal clearly demonstrate that there is a much larger variation in temperature between seasons in the North as compared to the South [39]. We thus hypothesized that microbial communities associated to *S*. *muticum* would show combined effects of spatio-temporal differences, but that these differences also depend on the seaweed structure/part examined. Next-generation sequencing of the variable regions V5-V7 of bacterial 16S rDNA genes was applied to characterize the diversity of associated microbiota and describe differences in microbial community structure.

## Methodology

### Study area and samples collection

**Fig 1:**
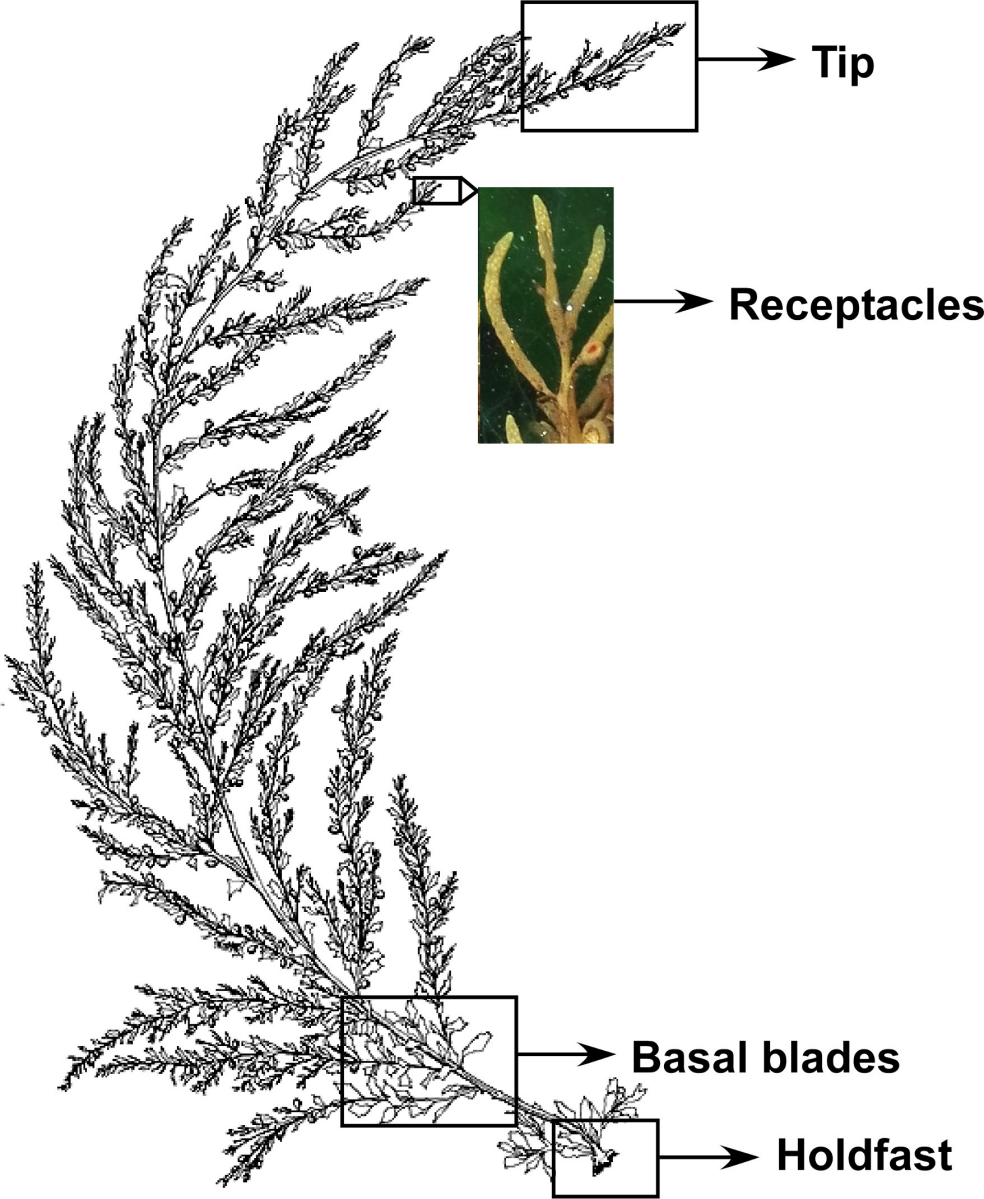
Schematic representation of *S*. *muticum* identifying the differentiated tissues considered in this study: Tip, receptacle, basal blades and holdfast.

Samples were collected in northern Portugal at Praia Norte (Viana do Castelo) and in southern Portugal at Praia do Queimado (Porto Covo) in September 2013, as well as in March, July (Porto Covo) & August (Viana do Castelo) 2014. The tissues, pieces of 1–2 cm, collected constituted: the tip, basal blades and holdfast, as well as reproductive structures (receptacles) (Fig 1) collected in July in Porto Covo and in September & August in Viana do Castelo. Each tissue was sampled in triplicate, separated by several meters to cover local variation, at each location and month. Sampled individuals were haphazardly chosen from the more complete developed individuals of the population to assure the presence of as many different structures as available and standardize the developmental status among individuals. In addition, sediments and water samples were collected. For sediments, three replicates were sampled in each month apart from September in Viana do Castelo (only 2 replicates). Seawater samples were also represented by three replicates, except September (in both locations), when only 2 replicates were available, and August in Viana do Castelo, where only 1 sample was collected. In the field, seawater microbiome was sampled by filtering 0.5 L of seawater over a 0.2 μm filter. All samples, seaweed and environmental, were preserved in the field directly upon collection in Xpedition lysis buffer (Zymo Research, California, USA). Overall, 94 samples were collected across both locations: 47 from Porto Covo (Southern) and 47 from Viana do Castelo (Northern). No collection permits were required as no individuals were sampled. The model species is not a protected or endangered species.

### High-throughput sequencing of the microbiome

Epiphytic and endophytic bacteria were extracted from differentiated structures (holdfast, basal blades, tip blades and receptacles) of *S*. *muticum* (Fig 1), seawater and sediments using MoBio PowerSoil DNA Isolation Kit in following the manufacturer protocol. The total 16S rRNA was amplified using the universal primers 27F and 1492r with the following changes to the original protocol [40]: following the initial denaturation step at 95°C for 2min, conditions constituted 35 cycles of denaturation at 95°C for 20s, annealing at 55°C for 20s, and extension at 72°C for 90s. The final extension was at 72°C for 3min. The 25 μl reaction mixture contained 250 μM dNTPs, 0.6 μM of each primer, 1 × 2PCR buffer mix, 2 μl of template DNA (with a final concentration of about 10 ng μl–1), and 0.3 μl of Taq polymerase (Advantage R2 Clontech). PCR products were cleaned using the ExoFastAP enzyme according to the manufacturer protocol (Thermo Scientific) and amplified DNA was sent to Molecular Research (MR DNA), Shallowater, Texas where a nested–PCR was implemented before the sequencing. Modified 8 bp key–tagged primer 799F along with the reverse primer 1193R (fragment ~ 400 bp), which avoid chloroplast cross amplification [41], were applied and PCR conditions constituted: 95°C for 3 min, 10 cycles of 95°C for 20 s, 50°C for 30 s, 72°C for 30 s, and a final elongation of 72°C for 3 min. Samples were pooled together in equal proportions based on their molecular weight and DNA concentrations and purified using calibrated Ampure XP beads. DNA libraries were prepared applying the Illumina TruSeq DNA library preparation protocol and paired–end (2 x 250 bp) sequencing performed at MR DNA (www.mrdnalab.com, Shallowater, TX, USA) on a MiSeq following the manufacturer’s guidelines.

### Sequence analysis and bioinformatics

The microbial community analysis was implemented using the Quantitative Insights into Microbial Ecology (QIIME version 1.8.0) program [42]. Sequences were screened and filtered for a minimum read length of 350 bp and less than 2 undetermined nucleotides. The filtered dataset, comprising only high-quality sequences, was applied to a conservative chimera detection filter using the ChimeraSlayer method [43]. Selected high quality chimera-free sequences were clustered into Operational Taxonomic Units (OTUs) within reads using the UCLUST algorithm [44] with a pairwise identity threshold of 0.97. Representative sequences for each OTU were selected using the “most-abundant” method and OTU sequence alignment was implemented with the Pynast tool [42]. The Ribosomal Database Project (RDP) classifier [45] was applied for taxonomic assignment with a 95% confidence threshold. To assign each OTU to the closest matching described taxon, the search was performed against the Greengenes reference database (version 12_10) [46] with a maximum e-value to record an assignment of 0.001. Sequences with the best match for eukaryotes (i.e. chloroplasts & mitochondria), rare OTUs (i.e. singletons & doubletons), and unassigned sequences were removed from the OTU table in the downstream analysis. The filtered rarefied OTU table was applied to calculate alpha diversity statistics, including the Chao I richness estimates [47], the observed number of OTUs, and the Shannon index, using QIIME software [42]. Permutational multivariate analysis of variance (PERMANOVA) was conducted to test for spatial and temporal differences in the microbiomes of *S*. *muticum*. To visualize dissimilarity between samples, Canonical Analysis of Principal coordinates (CAP) plots were constructed using the interaction among locationXtissueXseason as *a priori* factors. Bacterial contributions to similarity and dissimilarity between microbial communities was assessed by SIMPER analysis. All statistical analysis mentioned above were implemented using the software PRIMER-E+PERMANOVA v.6 [48, 49]. For bacterial groups, which showed notable differences in abundance between seasons, two-way analyses of variance (ANOVA) were implemented (with the preliminary tests for normality and homogeneity of variances).

## Results

**Fig 2:**
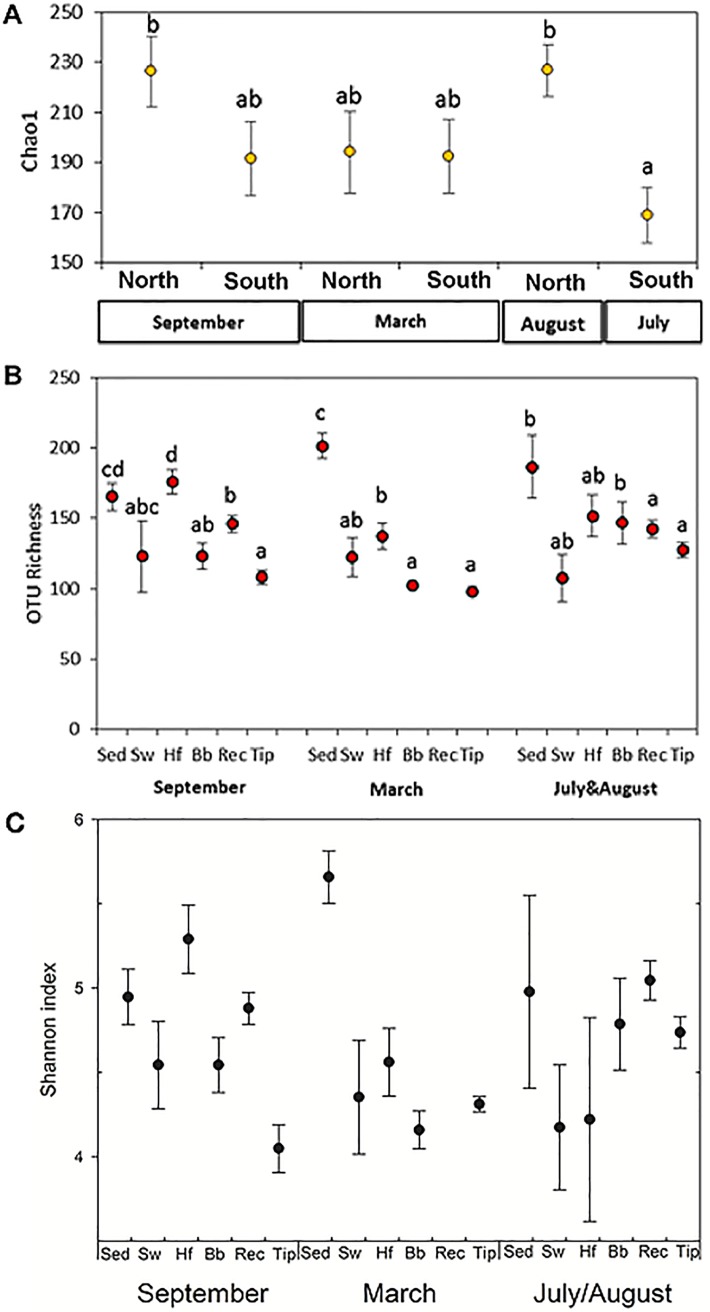
Alpha-diversity of bacterial communities. Chao 1 (A), OTU richness (B) and Shannon index (C) alpha diversity measures of bacterial communities associated to *S*. *muticum* across seasons at its northern (VC: Viana do Castelo) and southern distribution in Portugal (PC: Porto Covo). Differentiated are the environmental samples of sediments (color circle) and seawater (empty square), and the seaweed structures holdfast (rombe), basal blades (color square), receptacles (color triangle), and tip (empty circle). Values are means ± standard error (n = 3).

Rarefractioning to 2719 high quality sequences per sample based on the lowest number of available reads of a sample, resulted in 255,586 sequences in total that were used and corresponded to 52,109 OTUs. Overall, diversity was 1.2 times higher in northern compared to southern Portugal when expressed as Chao1 (PERMANOVA, P = 0.001; Fig 2A) and OTU Richness (PERMANOVA, P = 0.001, Fig 2B), except for Chao1 in March, and all months for the Shannon index (Fig 2C). Differences among tissues were also revealed for Chao1 (PERMANOVA, P = 0.001, Fig 2C), OTU Richness (PERMANOVA, P = 0.001, Fig 2B), and Shannon index (PERMANOVA, P = 0.009, Fig 2C). However, an interaction was observed between month and seaweed structure for OTU Richness (PERMANOVA, P = 0.035; Fig 2B). Overall, holdfasts were characterized by the highest bacterial diversity among all seaweed structures examined, after sediments (Fig 2B and 2C). Bacterial diversity tended to decrease towards the apical seaweed tissues with the lowest diversity associated to the tips (Fig 2B and 2C).

**Fig 3:**
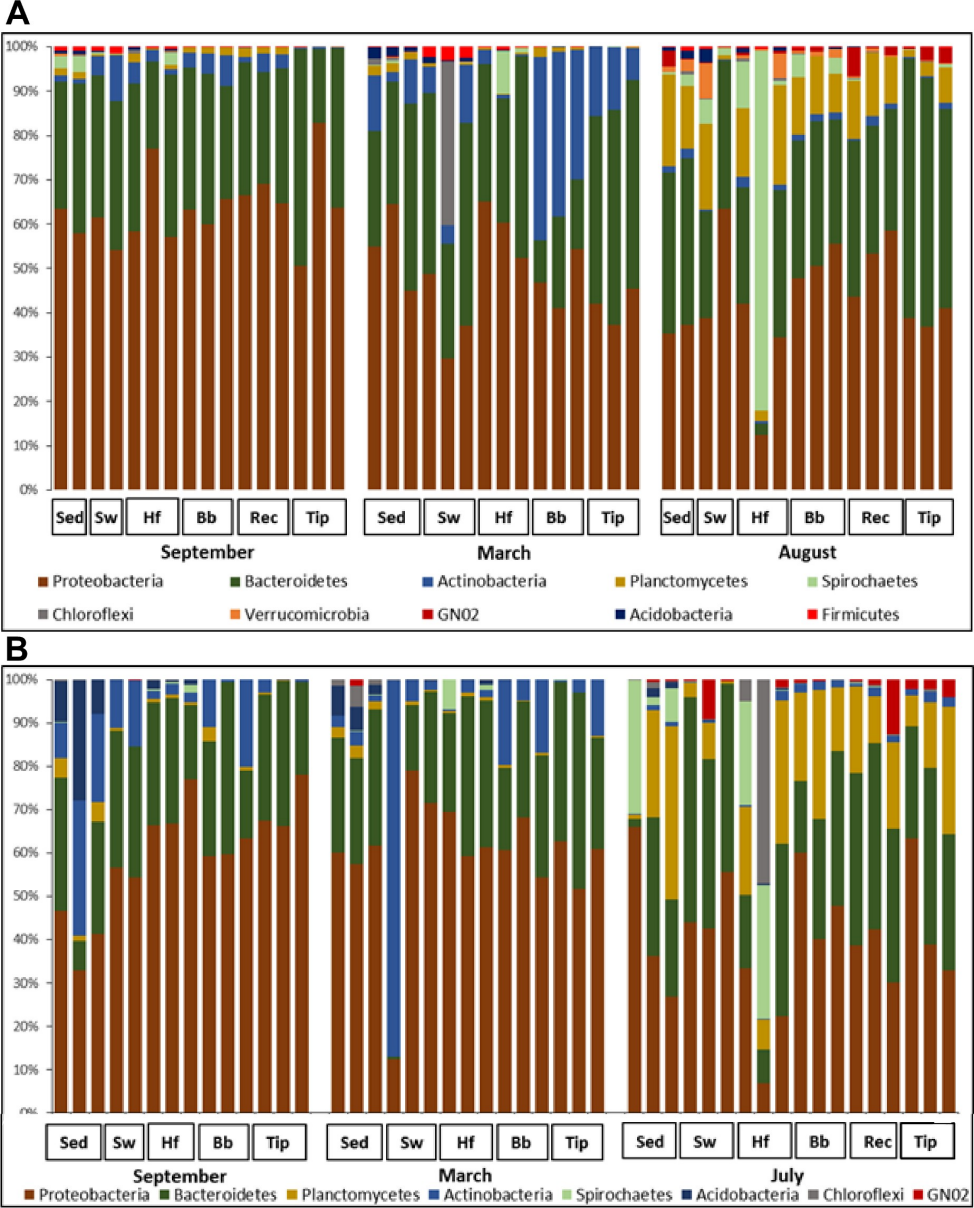
Distribution of bacteria phyla associated with *Sargassum muticum*. Relative distribution of the bacteria phyla associated with different structures of the brown seaweed *Sargassum muticum*, sediments (Sed) and surrounding seawater (SW) in northern (Viana do Castelo (A)) and southern (Porto Covo (B)) Portugal in September, March and July/August. Tissues are holdfast (Hf), basal blades (Bb), receptacle (Rec) and tip (Tip).

Overall, 794 bacterial genera were distributed across 137 classes from 52 phyla. In the South, 623 genera were distributed across 104 classes from 40 phyla, while in the North 682 genera were distributed across 124 classes from 52 phyla. September-March bacterial communities associated to *S*. *muticum* were dominated by *Proteobacteria* (60.5%; *Alpha* 36%, *Gamma* 17.9% & *Delta* 4.9%), *Bacteroidetes* (29.9%; *Flavobacteriia* 21.1% & *Saprospirae* 4.7%), and *Actinobacteria* (7.3%; *Acidimicrobiia* 7.2%) (Fig 3). Although *Proteobacteria* (40%; *Alpha* 18.3% & *Gamma* 17.5%) and *Bacteroidetes* (31.4%; *Flavobacteriia* 15.5% & *Saprospirae* 11.7%) are still major contributors to the bacterial communities in summer (July-August), a major change was reported with a high prevalence of *Planctomycetes* (14.5%; *Plantomycetia* 11.4%) and *Spirochaetes* (6.5%; *Spirochaetes* 6.5%) (Fig 3).

**Fig 4:**
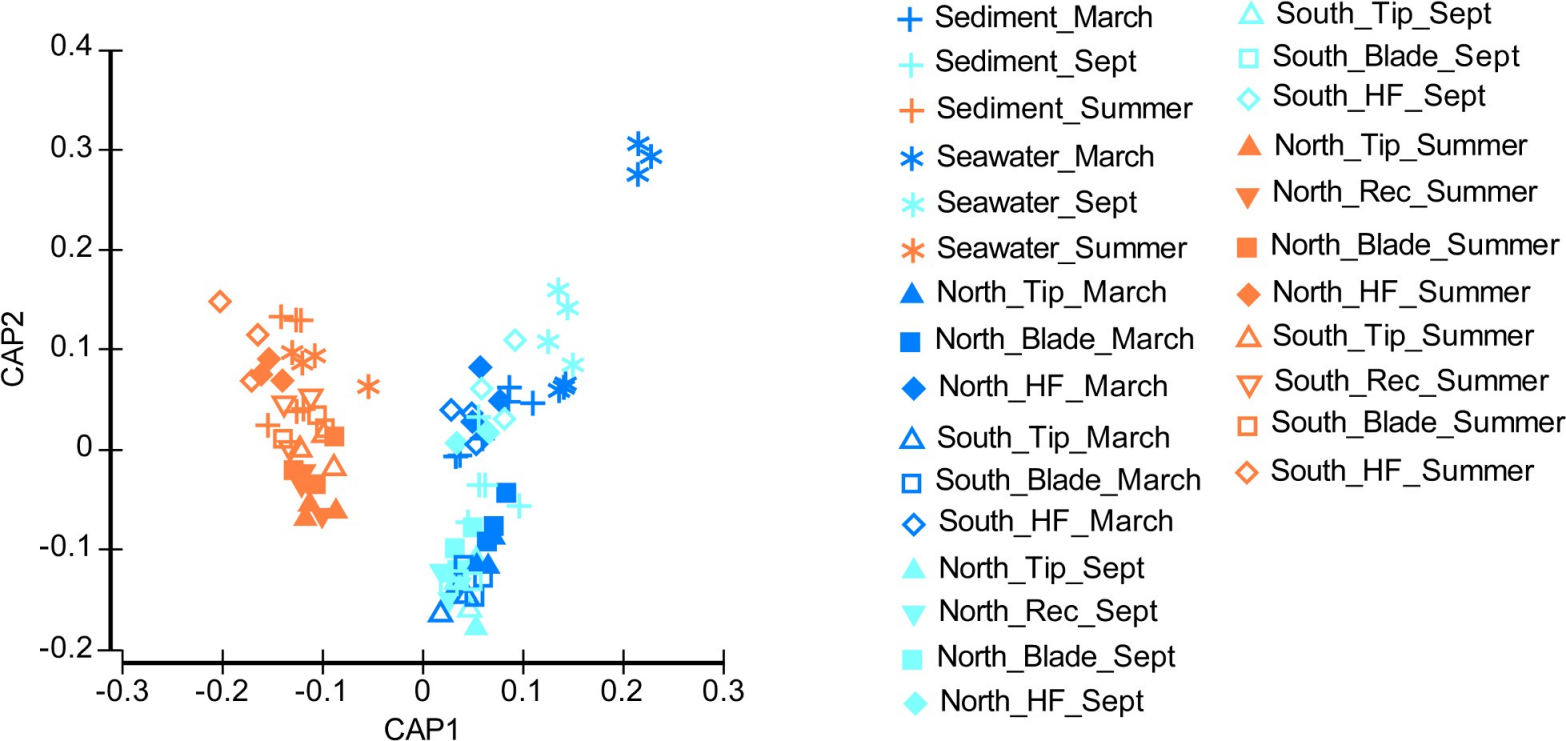
Structure of microbial communities associated with *Sargassum muticum*. Canonical Analysis of Principal coordinates of the bacterial communities associated with *Sargassum muticum*, sediments (+) and seawater (*) samples collected in southern (Porto Covo) and northern (Viana do Castelo) Portugal in September (light blue), March (dark blue) and July-August (orange). Distinguished structures are holdfast (HF) (diamond), basal blades (square), receptacle (Rec) (down facing triangle) and tip (up facing triangle).

Bacterial community structure associated with different *S*. *muticum* structures, sediments and seawater differed structurally in the two regions (North and South), among September, March and July-August (PERMANOVA, p = 0.006, Table A in S1 File), as shown by the canonical analysis of principle coordinates (Fig 4). Overall, *S*. *muticum* structures, sediment and seawater had different communities associated to them. Across structures, receptacles and basal blades had marginally more similar communities (PERMANOVA pairwise comparison P<0.053) as compared to holdfasts which harbored the most distinct associated community (Fig 4).

Microbial communities associated with seaweed structures showed temporal differences among all sampling times in the North (PERMANOVA P = 0.0001), but not in the South between September and March (PERMANOVA P = 0.1766; Fig 4). In contrast, environmental (sediments and seawater) microbial communities did not show temporal variations depending on location (PERMANOVA P = 0.070). Temporal variation in community structure was dependent on the seaweed structure examined (PERMANOVA P = 0.001), only holdfast associated communities did not differ among all sampling times (P = 0.188).

The most overall differences in community structure between summer and March—September constituted a decrease in *Proteobacteria* by 20.5% and *Actinobacteria* by 7.4%, as well as an increase in *Planctomycetes* by 13.8% (Fig 3). The lower abundances of *Proteobacteria* in summer, compared to September, occurred mostly due to the significant decline in abundance of an unidentified *Rhodobacteraceae* (within the tip, receptacles & basal blades) and *Loktanella* (within the tip, receptacles, basal blades and holdfast) both belonging to the order *Rhodobacterales*. Strongly increased abundance of *Planctomycetes* in summer, compared to September and March, occurred mostly due to an increase of an unidentified *Pirellulaceae* associated with receptacles, basal blades and holdfast.

Overall, in September-March, the most abundant bacteria associated with *S*. *muticum* were unidentified *Rhodobacteraceae* (9.9%), *Loktanella* (8.6%), unidentified *Flavobacteriaceae* (8.5%) and unidentified *Hyphomonadaceae* (5.4%). In contrast, in July-August, the most abundant bacteria were unidentified *Saprospiraceae* (10.2%), unidentified *Pirellulaceae* (10%), unidentified *Gammaproteobacteria* (9.4%), unidentified *Flavobacteriaceae* (7%), *Spirochaeta* (6.4%), and unidentified *Rhodobacteraceae* (5.5%).

Community structure associated with *S*. *muticum* was different between southern and northern Portugal (PERMANOVA, P = 0.001). The main differences were due to the prevalence of unidentified *Pirellulaceae (Pirellulales)* and unidentified *Gammaproteobacteria* in the South (contributing 1.71% and 1.67% to dissimilarity respectively; SIMPER), and higher abundance of *Loktanella (Rhodobacterales)* and unidentified *Saprospiraceae (Saprospirales)* in the North (contributing 1.65% and 1.62% to the dissimilarity respectively; SIMPER). The North-South differences in community was reflected in the communities associated with sediment (P = 0.0012) and each seaweed structure (P = 0.0004–0.0014), except holdfast (P = 0.4647) and seawater (P = 0.0754), where no such difference was detected.

**Fig 5:**
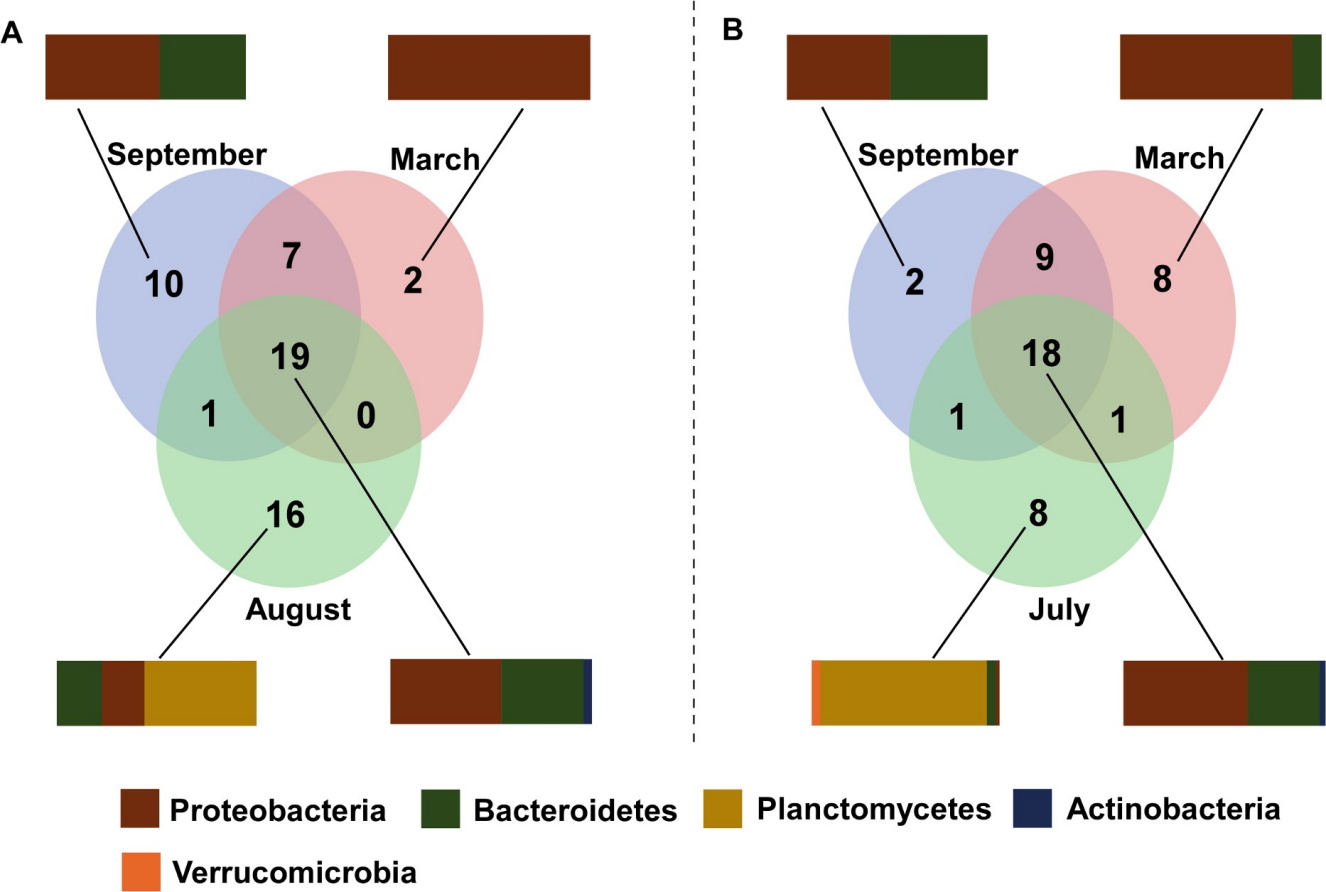
*Sargassum muticum* consistent bacterial genera across structures. Venn diagram representing the number of bacterial genera present across all *S*. *muticum* structure samples in North (A) and South (B) Portugal in different months. Vertical bars show phylum composition of each month (non-shared genera) and shared among all months.

Overall, 18 and 19 bacterial genera in southern and northern Portugal, respectively, were present in all tissues at all times, constituting *Alphaproteobacteria*, *Gammaproteobacteria*, *Flavobacteria*, *Saprospirae* and *Acidimicrobia* (Fig 5). These communities were very similar between the locations (Table B in S1 File). *Proteobacteria* and *Bacteroidetes* constituted the seasonally-independent bacterial phyla observed in all months sampled, *Planctomycetes* were unique to *S*. *muticum* only in Summer.

Temporal differences in communities for each seaweed structure were seldom at the southern (2 out of 9 pairwise comparisons), but common at the northern location (7 out of 10 pairwise comparisons; Table C in S1 File).

**Fig 6:**
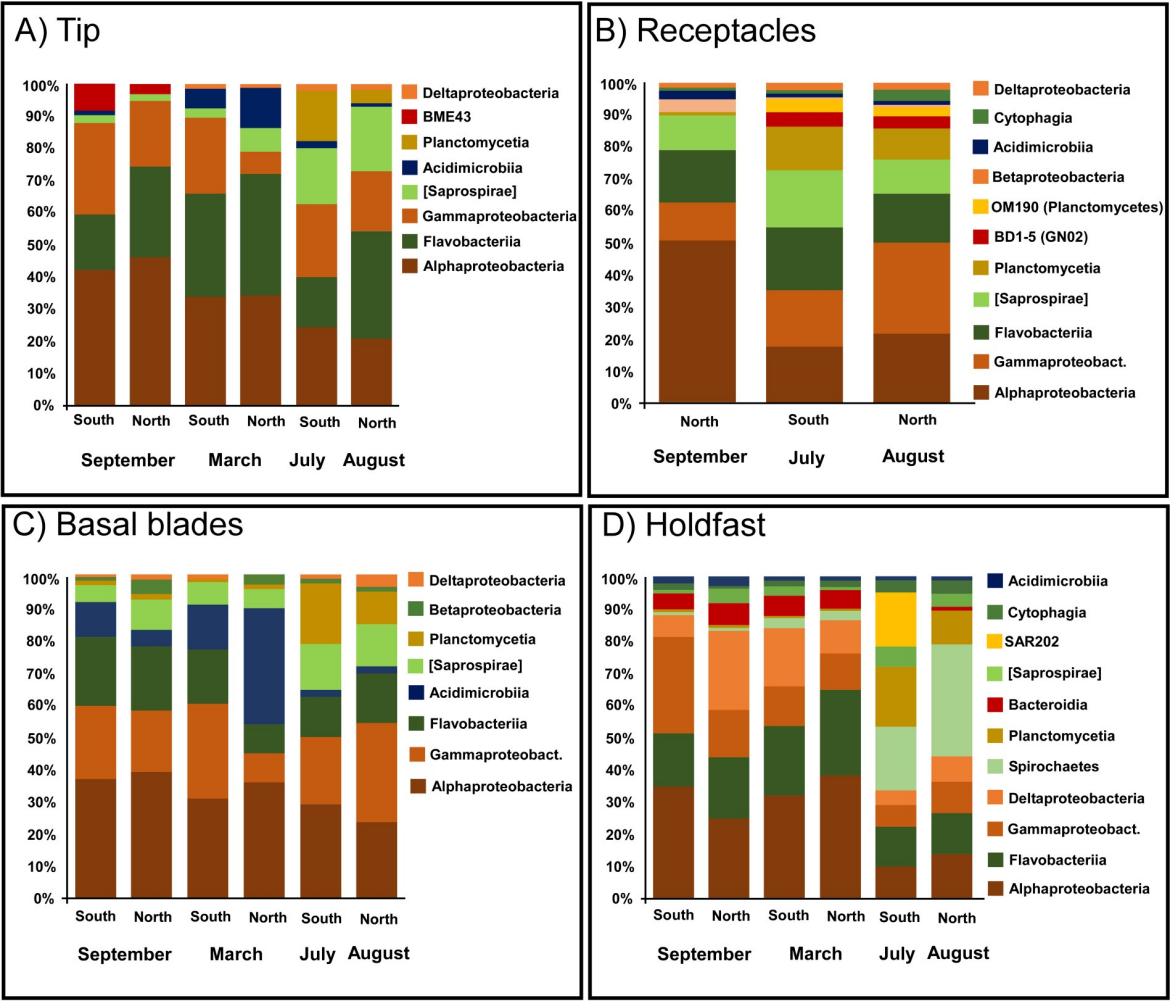
Most abundant bacterial classes associated with the different types of *S*. *muticum* tissues across seasons between North and South Portugal. A) Tips; B) Receptacles; C) Basal blades; D) Holdfasts.

Tip associated communities changed over all sampled seasons, but differently so between the northern and southern *S*. *muticum*, with in the South only temporal differences in Summer, while in the north temporal differences occurred across all sampling times (Table C in S1 File). Between September and March, an unidentified *Acidimicrobiales (Acidimicrobiia)* and *Loktanella (Alphaproteobacteria)* contributed most (5.68% and 4.6% respectively) to the dissimilarity in the North (43.04% average dissimilarity, SIMPER), while an unidentified *Rhodobacteraceae (Alphaproteobacteria)* contributed most (3.66%) to the dissimilarity in the South (40.98% average dissimilarity, SIMPER) (Fig 6). Between March and summer, an unidentified *Acidimicrobiales (Acidimicrobia)* and *Tenacibaculum (Flavobacteria)* contributed most to the dissimilarity (4.77% and 3.81% respectively) in the North (47.74% average dissimilarity, SIMPER), while an unidentified *Pirellulaceae (Planctomycetia)* contributed most (4.58%) to the dissimilarity in the South (52.10%) (see Fig 6 for relative abundances). Between summer and September, *Loktanella (Alphaproteobacteria)* and an unidentified *Saprospiraceae* the North (45.64% average dissimilarity, SIMPER), while *Glaciecola (Gammaproteobacteria)* and an unidentified *Pirellulaceae (Planctomycetia)* contributed most to the dissimilarity (4.34% and 4.22% respectively) in the South (55.64% average dissimilarity, SIMPER) (Fig 6).

Receptacles were only found during August (North) and September (North and South). In the North, differences were detected between summer and September (P = 0.019, average dissimilarity 39.65%, SIMPER) with an unidentified *Pirellulaceae (Planctomycetia)* and *Loktanella (Alphaproteobacteria)* contributing most to the dissimilarity, 3.48% and 3.41% respectively (Fig 6).

Microbial communities associated with basal blades showed differences between months only at the northern location, across all sampling times (Table C in S1 File). Between Summer and September (average dissimilarity 39.45%, SIMPER) an unidentified *Pirellulaceae (Planctomycetia)* contributed most (3.45%) to the dissimilarity, while between March and Summer (average dissimilarity 54.01%, SIMPER) most contribution to the dissimilarity was done by an unidentified *Acidimicrobiales* (4.38%) and an unidentified *JdFBGBact* (3.06%) both belonging to the class *Acidimicrobiia* (Fig 6). Between September and March (average dissimilarity 42.59%, SIMPER), an unidentified *Acidimicrobiales* contributed most (5.12%) to dissimilarity (Fig 6). No significant temporal differences in bacterial structure were revealed within the holdfast tissues.

Environmental communities showed temporal variation in community structure, but whereas in sediments structure differed among September, March and summer (P = 0.002–0.007), in seawater differences were restricted to between March and summer (P = 0.039).

## Discussion

Our results show that microbial communities associated to *Sargassum muticum* show temporal differences between September/March and July/August, but with different dynamics in northern and southern Portugal. In addition, these temporal and spatial differences depend on the seaweed structure examined.

The most pronounced temporal changes in microbial community were the significantly decreased abundance of *Proteobacteria* (by 20.5%) and *Actinobacteria* (by 7.4%) and increased abundance of *Planctomycetes* (by 13.8%) in summer. The summer decrease in *Proteobacteria* occurred mostly due to the significant decline in of unidentified *Rhodobacteraceae* (within the tip, receptacles & basal blades) and *Loktanella* (within the tip, receptacles, basal blades and holdfast). *Rhodobacterales* have been previously isolated from the seagrasses *Thalassia hemprichii* [50] and *Zostera marina* [51] and are primary colonizers of in marine surfaces with a known ability to fix nitrogen [52, 53]. Although not all members of the *Rhodobacteraceae* are considered pathogens, certain members are known to cause infections and disease (with the potential of getting more severe at increased temperatures) in *Fucus vesiculosus* [54] and *Delisea pulchra* [55]. *Loktanella* are known from various macroalgal species [2, 56, 57], including *Fucus vesiculosus* [21, 54], *Ulva australis* [30] and *Cystoseira compressa* [22]. Bacteria from this genus are highly adaptive and known for their capacity to utilize and rapidly metabolize organic carbon sources from seaweed exudates [20, 58, 59]. A significant increase of these bacteria was associated with the natural degradation of aged thalli in *Cystoseira compressa* during annual shedding in October [22]. Decrease in abundance of *Rhodobacteraceae* in response to elevated temperature levels was also observed in association with *Fucus vesiculosus* forma *mytili* [60]. The *S*. *muticum* material used in this study all seemed in good health and where not degrading and without signs of infection or disease.

Another important change was the summer increase of *Planctomycetes*, which occurred mostly due to the increase of unidentified *Pirellulaceae* from the order *Pirellulales* (within the holdfast, basal blades & receptacles). *Planctomycetes* are frequent associates of macroalgae [2, 9, 19, 30, 61]. They were reported in association with *Laminaria hyperborea* [62, 63], *Macrocystis pyrifera* [64], *Porphyra umbilicalis* [57], *Ulva australis* and *Delisea pulchra* [29], *Fucus vesiculosus*, *Gracilaria vermicuphylla* and *Ulva intestinalis* [21]. *Planctomycetes* are known for their ability to mineralize organic into inorganic compounds matching nutritional requirements of macroalgae [61]. *Planctomycetes* are also proposed to degrade algal polymers and important contributors to the global nitrogen cycle [65]. Because *Planctomycetes* contain a high number of sulfatases genes, they could participate in the degradation of sulfated polysaccharides produced by *S*. *muticum* [66, 67]. Although *Planctomycetes* are known to be abundant on macroalgae [61, 68], their relative abundances vary substantially between seasons and seaweed species [21]. The summer increase of *Planctomycetes* is in line with the study on *Laminaria hyperborea*, where the abundance of this bacterial phylum was minimal in September and maximal in July [19]. It has been proposed that without the seaweed chemical defense, *Planctomycetes* could lose their competitiveness over other bacteria, resulting in the low abundance observed in September [62].

### Temporal independent bacterial communities

Overall, *Proteobacteria* and *Bacteroidetes* constituted the most abundant bacterial phyla associated with *S*. *muticum* which was consistent with previous studies in other seaweeds [19, 21, 22, 24, 25, 30]. *Proteobacteria* (*Alpha*, *Gamma & Delta*), *Bacteroidetes* and *Actinobacteria* were prevalent in association with Baltic and North Sea *Fucus vesiculosus* [54, 60, 69], *Cystoseira compressa* [22] and *Macrocystis pyrifera* [64]. Prevalence of *Proteobacteria*, *Bacteroidets* and *Actinobacteria* over the year indicates of their seasonal independence (consistent with results of [21]) and temporal adaptation, as well as an important role towards *S*. *muticum* and functioning of its associated bacterial community.

### Differences between North and South

*Loktanella* (*Alphaproteobacteria*) and unidentified *Saprospiraceae* (*Bacteroidetes*) were more prevalent at the northern, while unidentified *Pirellulaceae* (*Planctomycetes*) and unidentified *Gammaproteobacteria* were more abundant at the southern location. Between September-March and summer, *Loktanella* and unidentified *Gammaproteobacteria* exhibited significant decrease in abundance, while unidentified *Saprospiraceae* and unidentified *Pirellulaceae* showed significant increase. Overall, the effect of season on *S*. *muticum* associated microbiota was more important than the effect of geographic location (Fig 4). A possible explanation for these results might be that despite the temperature gradient between the two locations throughout the year, the difference in seawater temperature might not be sufficient to significantly re-organize and re-structure associated microbial communities.

### Temporal differences by tissue

The highest temporal changes in *S*. *muticum* microbiota were detected within the tip tissues. This could be due to the fact that tips are made of newly developed (annual) tissues and, as such, recently colonized as compared to older perennial holdfast tissues. Another major change were the significant differences within the basal blades among the sampled months and within the receptacles between September and summer in the North, but not in the South. This could be due to the higher variation in water temperature in the North of Portugal compared to the South. In addition, for receptacles, reproductive activity of *S*. *muticum* in northern Portugal is finished by September and re-organization of associated microbiota is likely to have taken place before (notably when reproduction occurs between April and August).

### Possible sources of bacteria

Variation of bacterial communities associated with *S*. *muticum* could be explained by the variation in abundance of bacteria in the environment, from where they could be acquired, at the particular season [70]. The differences in composition between bacterial communities associated with *S*. *muticum* and seawater maybe due to the influence of the chemistry of the seaweed surface tissue (i.e. effect of seaweed metabolites on bacterial growth and attachment), which selectively attracts specific bacteria forming microbial composition driven by seaweed exudates [56, 64]. Pre-existing bacterial communities on the seaweed surface may affect the ability of settling bacteria to attach [70].

In this study, the abundance of unidentified *Rhodobacteraceae* was generally the highest in the seawater (followed by *S*. *muticum*) and the lowest in the sediments, indicating that these bacteria could be acquired by the seaweed from the surrounding water. Similarity in patterns between unidentified *Rhodobacteraceae* observed within *S*. *muticum* and in the seawater suggest that these bacteria could follow the seasonal pattern of its availability in the water column in response to variations in temperature, light availability and other factors. In contrast, *Loktanella* was more abundant in association with *S*. *muticum* than in the seawater across all seasons and locations, and least abundant in the sediments. An increased abundance of *Loktanella* during September-March could be linked to the fact that these bacteria could be attracted by seasonal changes in algal exudates at these months. The abundance of unidentified *Pirellulaceae* was higher in the sediments than within *S*. *muticum* and the lowest in the seawater across all seasons and locations, indicating that these bacteria could also be acquired by the seaweed from the sediments.

## Conclusion

In this study, we demonstrated that bacterial communities associated with *S*. *muticum* experience significant temporal shifts as well as variation between geographic locations. The temporal effect was reflected in significant abundance of unidentified *Rhodobacterales* and *Laktonella* in September-March and substantial prevalence of unidentified *Pirellulales* in summer. Such changes within *S*. *muticum* microbiota could be related to the seaweed productivity as temporally changing algal exudates attract different bacteria, which degrade algal polysaccharides and cell walls among other functions [62]. The temporal changes occurred mostly within the tip tissues and less within the basal blades and, possibly, receptacles. This could be related to the tips being younger compared to the other seaweed tissues and possibly under more direct colonization by bacteria from the surrounding environment. To what extend these re-organization and re-structuring of microbiota associated to *S*. *muticum* have potential consequences for the seaweed fitness and adaptation to environmental changes resulting in increased invasiveness [71] remains to be resolved.

## Supporting information

S1 File Table A. PERMANOVA results: Structure of bacterial communities associated with *S. muticum*, seawater and sediments (ti) at two locations (lo) and three months (mo). Table B. PERMANOVA Pair-wise tests between locations by month and seaweed structure of the interaction term ‘location x month x tissue’ of bacterial community structure associated with *S. muticum*, seawater and sediments at two locations and three months. Table C. PERMANOVA Pair-wise tests among months by location and seaweed structure of the interaction term ‘location x month x tissue’ of bacterial community structure associated with *S. muticum*, seawater and sediments at two locations and three months.
(DOCX)
