# Supplementary material for: Summer shifts of bacterial communities associated with the invasive brown seaweed Sargassum muticum are location and tissue dependent
Source: PLoS One. 2018 Dec 5;13(12):e0206734. doi: 10.1371/journal.pone.0206734 (PMC6281184; doi:10.1371/journal.pone.0206734)
Supplement: S1 File — Table A. PERMANOVA results: Structure of bacterial communities associated with S. muticum, seawater and sediments (ti) at two locations (lo) and three months (mo). Table B. PERMANOVA Pair-wise tests between locations by month and seaweed structure of the interaction term ‘location x month x tissue’ of bacterial community structure associated with S. muticum, seawater and sediments at two locations and three months. Table C. PERMANOVA Pair-wise tests among months by location and seaweed structure of the interaction term ‘location x month x tissue’ of bacterial community structure associated with S. muticum, seawater and sediments at two locations and three months. (DOCX) [file pone.0206734.s001.docx]

**Summer shifts of bacterial communities associated with the invasive brown seaweed *Sargassum muticum* are location and tissue dependent.**

Alexandra Serebryakova^1,2^, Tania Aires^1^, Frederique Viard^2^, Ester A. Serrão^1^, Aschwin H. Engelen^1^,

Supplementary Information

Supplementary Table A - **PERMANOVA results: Structure of bacterial communities associated with *S. muticum,* seawater and sediments (ti) at two locations (lo) and three months (mo).**

| **Source**  **df** **SS MS** **Pseudo-F P(perm) perms** |  |
| --- | --- |
| lo 1 3282.1 3282.1 4.0008 0.001 999 |  |
| mo 2 16513 8256.5 10.065 0.001 999 |  |
| ti 5 33975 6795.1 8.2831 0.001 999 |  |
| loxmo 2 3594 1797 2.1905 0.001 996 |  |
| loxti 5 6939 1387.8 1.6917 0.001 996 |  |
| Moxti 9 6681 1853.4 2.2593 0.001 995 |  |
| Loxmoxti 8 9016.7 1127.1 1.3739 **0.006** 998 |  |
| Res 61 50042 820.35 |  |
| Total 93 1.4764E5 |  |

Supplementary Table B –  **PERMANOVA Pair-wise tests between locations by month and seaweed structure of the interaction term ‘location x month x tissue’ of bacterial community structure associated with *S. muticum,* seawater and sediments at two locations and three months.**

North=Viana do Castelo; South=Porto Covo; rec=receptacles; hf=holdfast; sed=sediment; sw= seawater. P(MC) shows the p-value obtained from 999 Monte Carlo simulations. Significant comparisons: p<0.05, in bold.

| **Month** | **Structure** | **Groups** | **t** | **P(perm)** | **P(MC)** |
| --- | --- | --- | --- | --- | --- |
| March | sed | North *vs*. South | 1.5153 | 0.117 | 0.119 |
| March | blade | North *vs*. South | 1.6701 | 0.097 | 0.067 |
| March | hf | North *vs*. South | 0.7902 | 0.825 | 0.619 |
| March | tip | North *vs*. South | 1.6571 | 0.094 | 0.076 |
| March | sw | North *vs*. South | 1.3595 | 0.102 | 0.174 |
| Summer | sed | North *vs*. South | 1.1091 | 0.309 | 0.340 |
| Summer | rec | North *vs*. South | 1.3685 | 0.093 | 0.154 |
| Summer | blade | North *vs*. South | 1.4804 | 0.089 | 0.125 |
| Summer | hf | North *vs*. South | 0.9960 | 0.438 | 0.520 |
| Summer | sw | North *vs*. South | 1.1779 | 0.224 | 0.347 |
| Summer | tip | North *vs*. South | 1.8660 | 0.081 | 0.043 |
| September | sed | North *vs*. South | 2.2183 | 0.109 | **0.037** |
| September | blade | North *vs*. South | 1.3552 | 0.164 | 0.110 |
| September | hf | North *vs*. South | 1.0558 | 0.491 | 0.381 |
| September | sw | North *vs*. South | 1.1224 | 0.322 | 0.372 |
| September | tip | North *vs*. South | 1.3171 | 0.098 | 0.170 |

Supplementary Table C – **PERMANOVA Pair-wise tests among months by location and seaweed structure of the interaction term ‘location x month x tissue’ of bacterial communities structure associated with *S. muticum,* seawater and sediments at two locations and three months.**

VC=Viana do Castelo; PC=Porto Covo; rec=receptacles; hf=holdfast; sed=sediment; sw= seawater. P(MC) shows the p-value obtained from 999 Monte Carlo simulations. Significant comparisons: p<0.05 in bold.

| **Location** | **Tissue** | **Groups** | **t** | **P(perm)** | **P(MC)** |
| --- | --- | --- | --- | --- | --- |
| PC | blade | March *vs*. Summer | 1.7339 | 0.110 | 0.063 |
|  |  | March *vs*. September | 1.1085 | 0.297 | 0.329 |
|  |  | Summer *vs*. September | 1.6790 | 0.114 | 0.057 |
| PC | hf | March *vs*. Summer | 1.4570 | 0.105 | 0.133 |
|  |  | March *vs*. September | 0.8743 | 0.801 | 0.574 |
|  |  | Summer *vs*. September | 1.7711 | 0.095 | 0.058 |
| PC | tip | March *vs*. Summer | 2.2234 | 0.110 | **0.022** |
|  |  | March *vs*. September | 1.1941 | 0.200 | 0.269 |
|  |  | Summer *vs*. September | 2.1224 | 0.094 | **0.029** |
| VC | rec | Summer *vs*. September | 2.1926 | 0.115 | **0.019** |
| VC | blade | March *vs*. Summer | 2.6980 | 0.099 | **0.012** |
|  |  | March *vs*. September | 2.4515 | 0.117 | **0.013** |
|  |  | Summer *vs* September | 2.0846 | 0.080 | **0.019** |
| VC | hf | March *vs*. Summer | 1.7134 | 0.097 | 0.052 |
|  |  | March *vs*. September | 1.2519 | 0.106 | 0.223 |
|  |  | Summer *vs*. September | 1.5290 | 0.095 | 0.121 |
| VC | tip | March *vs*. Summer | 2.8434 | 0.086 | **0.011** |
|  |  | March *vs*. September | 2.0498 | 0.111 | **0.031** |
|  |  | Summer *vs*. September | 2.1540 | 0.105 | **0.017** |
